# Primary tumor location affects recurrence-free survival for patients with colorectal liver metastases after hepatectomy: a propensity score matching analysis

**DOI:** 10.1186/s12957-020-01875-y

**Published:** 2020-05-18

**Authors:** Yuanping Zhang, Yongjin Wang, Yichuan Yuan, Jiliang Qiu, Yuxiong Qiu, Wei He, Yun Zheng, Zhiqiang Wang, Yangkui Gu, Zhenhai Lu, Gong Chen, Peirong Ding, Xiaojun Wu, Zhizhong Pan, Desen Wan, Yuhong Li, Ruihua Xu, Yunfei Yuan, Binkui Li

**Affiliations:** 1 Department of Liver Surgery, Sun Yat-sen University Cancer Center, 651 Dongfeng Road East, Guangzhou, 510060 China; 2 State Key Laboratory of Oncology in South China, Sun Yat-sen University Cancer Center, Guangzhou, China; 3 Collaborative Innovation Center for Cancer Medicine, Guangzhou, China; 4 Department of Medical Oncology, Sun Yat-sen University Cancer Center, Guangzhou, China; 5 Microinvasive Interventional Department, Sun Yat-sen University Cancer Center, Guangzhou, China; 6 Department of Colorectal Surgery, Sun Yat-sen University Cancer Center, Guangzhou, China

**Keywords:** Colorectal cancer, Liver metastases, Location, Prognosis

## Abstract

**Background:**

Whether primary tumor location of colorectal cancer (CRC) affects survival of patients after resection of liver metastases remains controversial. This study was conducted to investigate the differences in clinicopathological characteristics and prognosis between right-sided CRC and left-sided CRC patients with liver metastases after hepatectomy.

**Methods:**

From 2002 to 2018, 611 patients with colorectal liver metastases (CRLM) who underwent hepatectomy at our center were reviewed. Primary tumors located from the cecum to transverse colon were defined as right-sided group (*n* = 141); tumors located from the splenic flexure to rectum were defined as left-sided group (*n* = 470). Patients were compared between two groups before and after a 1:1 propensity score matching (PSM) analysis.

**Results:**

Before PSM, median survival time and 5-year overall survival (OS) rate in right-sided group were 77 months and 56.3%, and those in left-sided group were 64 months and 51.1%, respectively. After PSM, median survival time and 5-year OS rate in right-sided group were 77 months and 55.9%, and those in left-sided group were 58.8 months and 47.3%, respectively. The OS rates did not differ between two groups before and after PSM (*P* = 0.575, *P* = 0.453). However, significant different recurrence-free survival (RFS) rate was found before and after PSM between right-sided and left-sided group (*P* = 0.028, *P* = 0.003).

**Conclusions:**

Compared to patients with left-sided primary tumors, patients with right-sided primary tumors had a worse RFS but similar OS. Careful preoperative evaluation, intensive preoperative chemotherapy, and frequent follow-up to detect early recurrence might be justified for CRLM patients with right-sided primary tumors.

## Background

Colorectal cancer (CRC) is the third most commonly diagnosed cancer in the world [1]. In Asia, its incidence and mortality has been also on the rise over recent decades [2]. Colorectal liver metastases (CRLM) present in 20–25% of patients at the time of diagnosis and in approximately 60% of patients during the course of the disease [3]. Despite improvements in the comprehensive management of patients with CRLM in recent years, liver resection remains the most effective treatment with the potential for long-term survival and cure for CRLM patients [4].

In CRLM patients undergoing hepatic resection, many factors such as tumor size of liver metastases, tumor number, and serum carcinoembryonic antigen (CEA) level are associated with outcomes [5]. Primary tumor factors including lymph node status, pathology grade, and genetic status may also influence the survival [6, 7]. Besides survival, genetic status of primary tumor was also found to predict resection margin and pathologic response in CRLM patients treated with neoadjuvant chemotherapy [8, 9].

Primary tumor location has been reported as a potential prognostic factor in patients with CRC. There is growing evidence that patients with right-sided CRC tend to present with higher TNM stage, larger tumor size and worse outcomes than those with left-sided CRC [10, 11]. Genetic differences may account for distinct carcinogenesis and biological behavior and lead to worse prognosis in right-sided CRC patients [12, 13]. However, the value of primary tumor location in determining prognosis of patients with CRLM remains controversial, due to the contradicting results in existing studies [14, 15].

In this study, we aimed to explore the impact of primary tumor location of CRC on clinical characteristics and survival for CRLM patients undergoing hepatectomy. Propensity score matching (PSM) was used to strengthen causal arguments in observational study by reducing selection bias.

## Methods

### Patients and study design

All pathologically confirmed CRLM patients undergoing hepatectomy at Sun Yat-sen University Cancer Center between July 2002 and March 2018 were included. Demographic and clinicopathologic variables were collected with review of the medical record. To avoid the impact of different pathological type for prognosis, only pathological type of adenocarcinoma was included. All patients received primary tumor resection prior to or combined with hepatectomy. Exclusion criteria consisted of the following: Child-Pugh score of C, Eastern Cooperative Oncology Group-performance status (ECOG-PS) > 2, had double primary malignancies, lost in follow-up. According to the anatomical location, primary tumors located from the cecum to transverse colon were defined as right-sided group, and tumors located from the splenic flexure to rectum were defined as left-sided group. Differences in clinicopathological characteristics and prognosis between the two groups (left-sided group vs. right-sided group) were compared to determine whether the primary tumor location of CRC affecting survival of patients after resection of liver metastases. The primary endpoints of this study were overall survival (OS) and recurrence-free survival (RFS). The OS was defined as the time interval from liver resection to death from any cause or the last follow-up date. RFS was defined as the time interval from liver resection to disease recurrence, death from disease, or the last follow-up date.

Preoperative blood tests which included tumor markers were carried out within 2 weeks before resection of CRLM. Image to evaluate the resectability of liver metastases included magnetic resonance imaging (MRI) or computed tomography (CT). Intraoperative ultrasonography (US) was performed as conventional procedure to conduct radical resection of all tumors if possible. Definition of R0 resection is resection of liver lesions with clear histological margins, and non R0 (R1/R2) resection is resection with histological positive margins or residual lesions in intra or extrahepatic. Liver metastases diagnosed before, during, or within 3 months after colorectal resection is defined as synchronous metastases.

The clinical risk score (CRS) used in the study was an established risk score—the “Fong” score, which is consisting of five clinical factors, including primary lymph node metastasis, synchronous metastases, multiple liver tumors, tumor size over 5 cm, and carcinoembryonic antigen (CEA) over 200 ng/ml [5]. Each of 5 clinical factors is assigned 1 point. Patients with a CRS of 0–2 were classified into the low-risk subgroup, while patients with a CRS of 3–5 were classified into the high-risk subgroup.

### Follow-ups

All patients were followed up monthly in the first 3 months, every 3 months in the first 2 years, and every 3 to 6 months thereafter. Physical examination, blood tests, abdominal and pelvic US, or CT/MRI was used for the surveillance of recurrence as appropriate.

### Statistical analysis

In all patients, propensity score matching (PSM) was performed to reduce selection bias. Propensity scores were estimated using a logistic regression model based on age, gender, primary tumor lymph node status, time of liver metastases, preoperative CEA level, preoperative chemotherapy, number of liver metastases, and size of largest liver lesions. A 1:1 nearest neighbor matching without replacement was performed using a 0.2 caliper width. The potential residual imbalance after matching was tested through univariate (standardized mean difference [SMD] cutoff 0.25) and multivariate tests (Hansen-Bowers test, Iacus-King-Porro test). The resulting score-matched pairs were used in subsequent analyses as indicated. Consecutive data were presented as mean (square deviation, SD). Independent-sample *T* test, Chi-square test, or Fisher’s exact test was used for analyzing the differences in clinicopathological characteristics between two groups as appropriate. The OS and RFS curves were constructed by Kaplan–Meier method and compared with the log-rank test. Cox proportional hazard regression model was performed to identify the hazard ratio (HR) of prognostic factors. A *P* value less than 0.05 was regarded as statistically significant. All *P* value of statistical tests in the present study was two-sided. All statistical calculations were performed with the IBM SPSS Statistics 25.0 software package (SPSS Inc., Chicago, IL).

## Results

### Clinicopathological characteristics

Of the 611 patients, 141 (23.1%) had primary tumors located in the right-sided CRC, and 470 (76.9%) had primary tumors located in the left-sided CRC. Clinicopathological characteristics of the two groups are presented in Table 1. Compared to the left-sided group, the right-sided group tended to have larger tumor size in CRC (4.2 vs. 3.6 cm, *P* = 0.011) and less people underwent preoperative chemotherapy before hepatectomy (48.2 vs. 61.1%, *P* = 0.008). Other baseline parameters such as largest size of liver tumors, number, and distribution of liver metastases were comparable between the two groups.

**Table 1:** Baseline clinicopathological characteristics

Characteristics	Before PSM (***n*** = 611)				After PSM (***n*** = 254)			
	Right-sided group (***n*** = 141)	Left-sided group (***n*** = 470)	***P*** value	SMD	Right-sided group (***n*** = 127)	Left-sided group (***n*** = 127)	***P*** value	SMD
Mean age (± SD), years	57 (± 12)	55 (± 11)	0.313	− 0.099	56 (± 12)	54 (± 12)	0.381	0.086
Gender, *n* (%)			0.538	0.065			0.894	0.016
Male	92 (65.2)	321 (68.3)			84 (66.1)	86 (67.7)		
Female	49 (34.8)	149 (31.7)			43 (33.9)	41 (32.3)		
**Primary tumor characteristics**								
Chemotherapy prior to CRC resection			0.218				0.364	
Yes	37 (26.4)	156 (33.3)			35 (27.8)	43 (33.9)		
No	103 (73.6)	313 (66.7)			91 (72.2)	84 (66.1)		
Surgery of CRC and CRLM, n (%)			0.067				0.900	
Staged	69 (48.9)	271 (57.7)			62 (48.8)	64 (50.4)		
Combined	72 (51.1)	199 (42.3)			65 (51.2)	63 (49.6)		
Tumor size, mean (± SD), cm	4.2 (± 2.3)	3.6 (± 2.2)	0.011*		4.2 (± 2.3)	3.7 (± 2.4)	0.048*	
T stage, *n* (%)			0.524				0.841	
T1/T2	9 (6.4)	40 (8.5)			8 (6.3)	10 (7.9)		
T3/T4	118 (83.7)	393 (83.6)			114 (89.8)	112 (88.9)		
unknown	14 (9.9)	37 (7.9)			5 (3.9)	5 (3.9)		
N stage, *n* (%)			0.546	− 0.061			0.363	-0.058
N0	52 (39.7)	188 (40.0)			51 (40.2)	43 (33.9)		
N1/N2	79 (60.3)	249 (53.0)			76 (59.8)	84 (66.1)		
Unknown	0 (0)	33 (7.0)						
TNM stage, *n* (%)			0.441				0.348	
I	2 (1.4)	10 (2.1)			2 (1.6)	0 (0.0)		
II	7 (5.0)	43 (9.1)			7 (5.5)	9 (7.1)		
III	21 (14.9)	73 (15.5)			21 (16.5)	20 (15.7)		
IV	106 (75.2)	331 (70.4)			96 (75.6)	98 (77.2)		
Unknown	5 (3.5)	13 (2.8)			1 (0.7)	0 (0.0)		
Postoperative chemotherapy, *n* (%)			0.256				0.475	
Yes	103 (73.0)	366 (77.9)			91 (71.7)	97 (76.4)		
No	38 (27.0)	104 (22.1)			36 (28.3)	30 (23.6)		
**CRLM characteristics**								
Presentation of CRLM, *n* (%)			0.253	− 0.113			0.883	− 0.062
Metachronous	35 (24.8)	140 (29.8)			31 (24.4)	29 (22.8)		
Synchronous	106 (75.2)	330 (70.2)			96 (75.6)	98 (77.2)		
Preoperative chemotherapy, *n* (%)			0.008*	0.268			0.900	− 0.045
Yes	68 (48.2)	287 (61.1)			64 (50.4)	62 (48.8)		
No	73 (51.8)	183 (38.9)			63 (49.6)	65 (51.2)		
Preoperative CEA (μg/L), (> 200/≤ 200) [*n* (%)]	6/135 (4.3/95.7)	23/447 (4.9/95.1)	0.755	− 0.008	6/121 (4.7/95.3)	5/122 (3.9/96.1)	1.000	− 0.042
Preoperative CA19-9 (kU/L), , (> 35/≤ 35) [*n* (%)]	30/110 (21.4/78.6)	97/370 (20.8/79.2)	0.867		26/100 (20.6/79.4)	24/103 (18.9/81.1)	0.754	
Tumor size (cm), median (IQR)	3.0 (2.0–4.5)	2.8 (1.6–4.0)	0.095	− 0.164	3.0 (1.8–4.0)	3.0 (1.5–4.0)	0.944	− 0.013
Position, *n* (%)			1.000				0.196	
Unilobar	79 (56.4)	263 (56.6)			72 (57.1)	81 (65.3)		
Bilobar	61 (43.6)	202 (43.4)			54 (42.9)	43 (34.7)		
Number of tumors, *n* (%)			0.627	0.082			0.528	− 0.015
Multiple	79 (56.0)	275 (58.5)			73 (57.5)	67 (52.8)		
Single	62 (44.0)	195 (41.5)			54 (42.5)	60 (47.2)		
R0 resection, *n* (%)			1.000				0.886	
Yes	119 (84.4)	396 (84.4)			106 (83.5)	106 (84.1)		
No	22 (15.6)	73 (15.6)			21 (16.5)	20 (15.9)		
Postoperative chemotherapy, *n* (%)			0.455				1.000	
Yes	98 (69.5)	342 (72.8)			89 (70.1)	90 (70.9)		
No	43 (30.5)	128 (27.2)			38 (29.9)	37 (29.1)		
CRS score, *n* (%)			0.538				0.922	
1–2	90 (64.7)	312 (67.7)			88 (71.0)	88 (70.4)		
3–5	49 (35.3)	149 (32.3)			36 (29.0)	37 (29.6)		

*PSM* propensity score matching, *SD* standard deviation, *IQR* inter-quartile range, *T stage* tumor stage, *N stage* node stage, *CRLM* colorectal liver metastases, *CEA* carcinoembryonic antigen, *CA 19-9* carbohydrate antigen 19-9, *R0 resection* hepatectomy on patients with clear histological margins, *SMD* standardized mean differences

*P < 0.05

After PSM, 127 matched pairs were generated from the right-sided and left-sided groups. The standardized mean differences of included PSM factors were decreased (Table 1). Also, Hansen-Bowers test for global imbalance was not significant (*P* = 0.996), and the Iacus-King-Porro test showed that L1 was reduced in the matched sample, indicating the improvement of the overall balance (L1: before matching 0.929; after 0.899). The reduction of imbalance is pictured by histograms with overlaid kernel density estimates for SMD (Supplementary Figure 1).

### Survival analysis

**Fig. 1:**
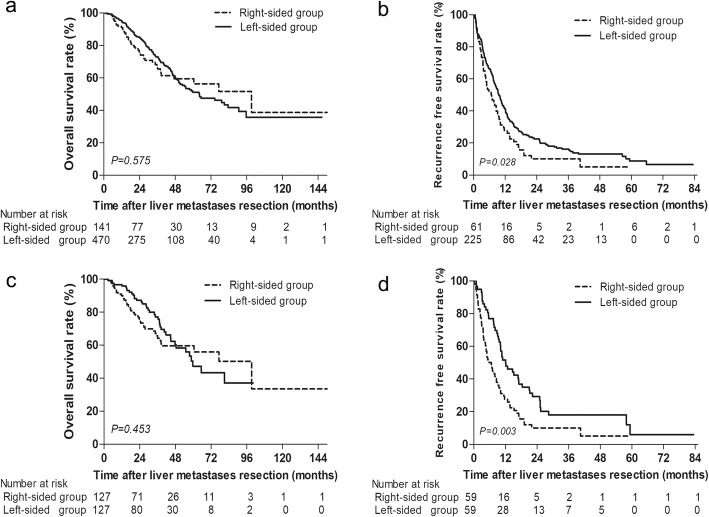
**a** Overall survival and **b** recurrence-free survival in CRLM patients stratified by CRC location. **c** Overall survival and **d** recurrence-free survival in CRLM patients stratified by CRC location after PSM

The average follow-up interval for all the included patients was 36.9 months (range 2.2–151.5 months). The median OS for the right-sided group and left-sided group were 77 months and 64 months, respectively. The 1-, 3-, and 5-year OS rates after CRLM resection in the right-sided group were 91.0%, 75.0%, and 56.3%, respectively, and 94.9%, 84.8%, and 51.1%, respectively, in the left-sided group (*P* = 0.575; Fig. 1a). The 1-, 3-, and 5-year RFS rates after R0 resection of liver metastases in the right-sided group were 27.8%, 10.1%, and 5.1%, respectively, and 40.9%, 22.6%, and 8.8%, respectively, in the left-sided group. Left-sided group have a significant better RFS rate than right-sided group (*P* = 0.028; Fig. 1b).

After PSM, the median OS time for patients in the right-sided group was 77 months and was 58 months in the left-sided group. Cumulative 1-, 3-, and 5-year OS rates were 89.2%, 64.2%, and 55.9%, respectively, in the right-sided group, compared to 95.9%, 75.7%, and 47.3%, respectively, in the left-sided group (*P* = 0.453; Fig. 1c). The median RFS for patients in the right-sided group was 5.8 months and was 10.9 months for patients in the left-sided group. Cumulative 1- and 3-year RFS rates were 25.9% and 10.1%, respectively, in the patients from the right-sided group, compared to 48.8% and 17.2%, respectively, in patients from the left-sided group (*P* = 0.003; Fig. 1d).

### Prognosis stratified by CRS score and tumor number

**Fig. 2:**
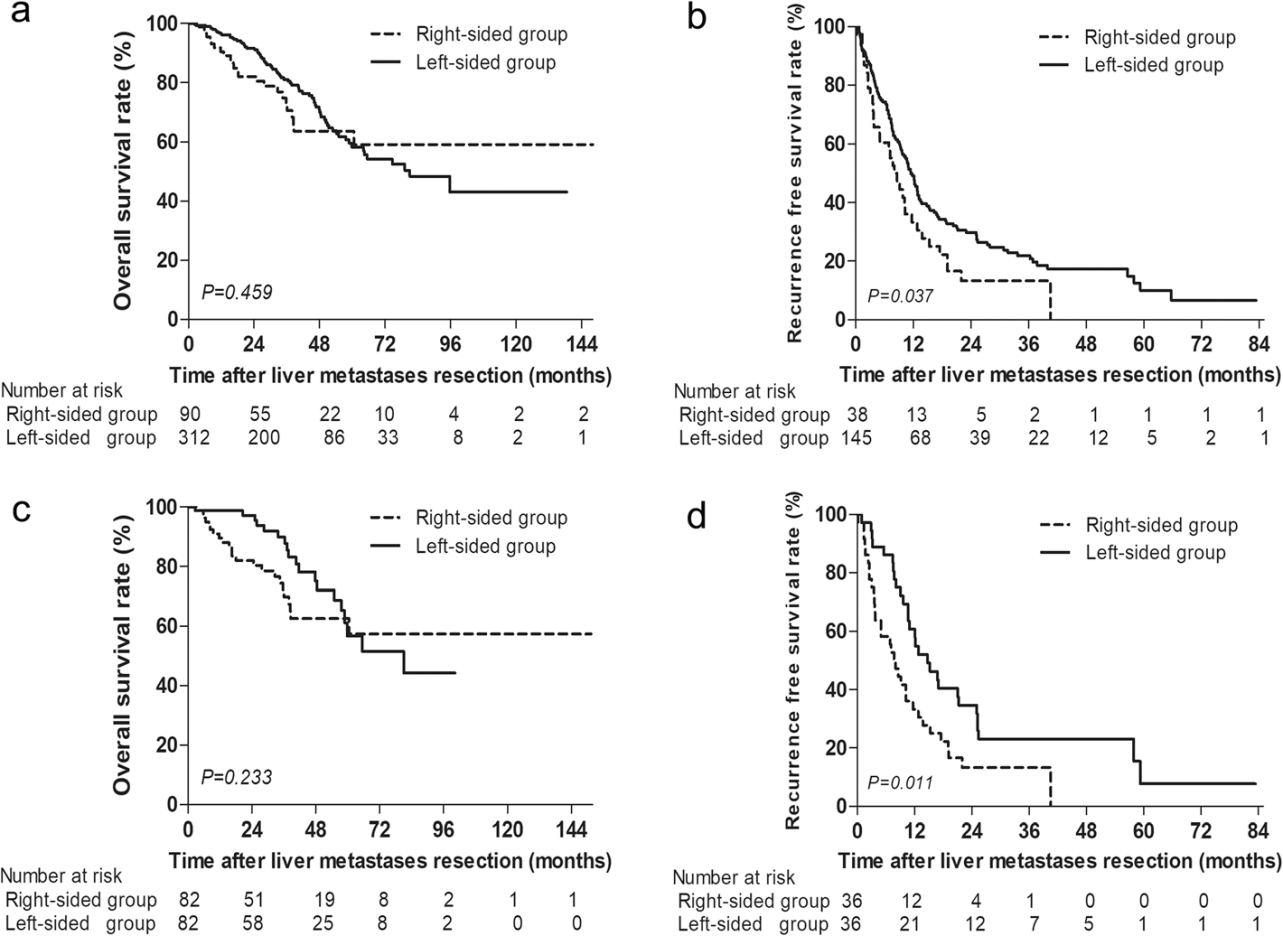
**a** Overall survival and **b** recurrence-free survival in CRLM patients stratified by CRC location with low CRS scores (score ≤ 2). **c** Overall survival and **d** recurrence-free survival in CRLM patients stratified by CRC location with low CRS scores after PSM

**Fig. 3:**
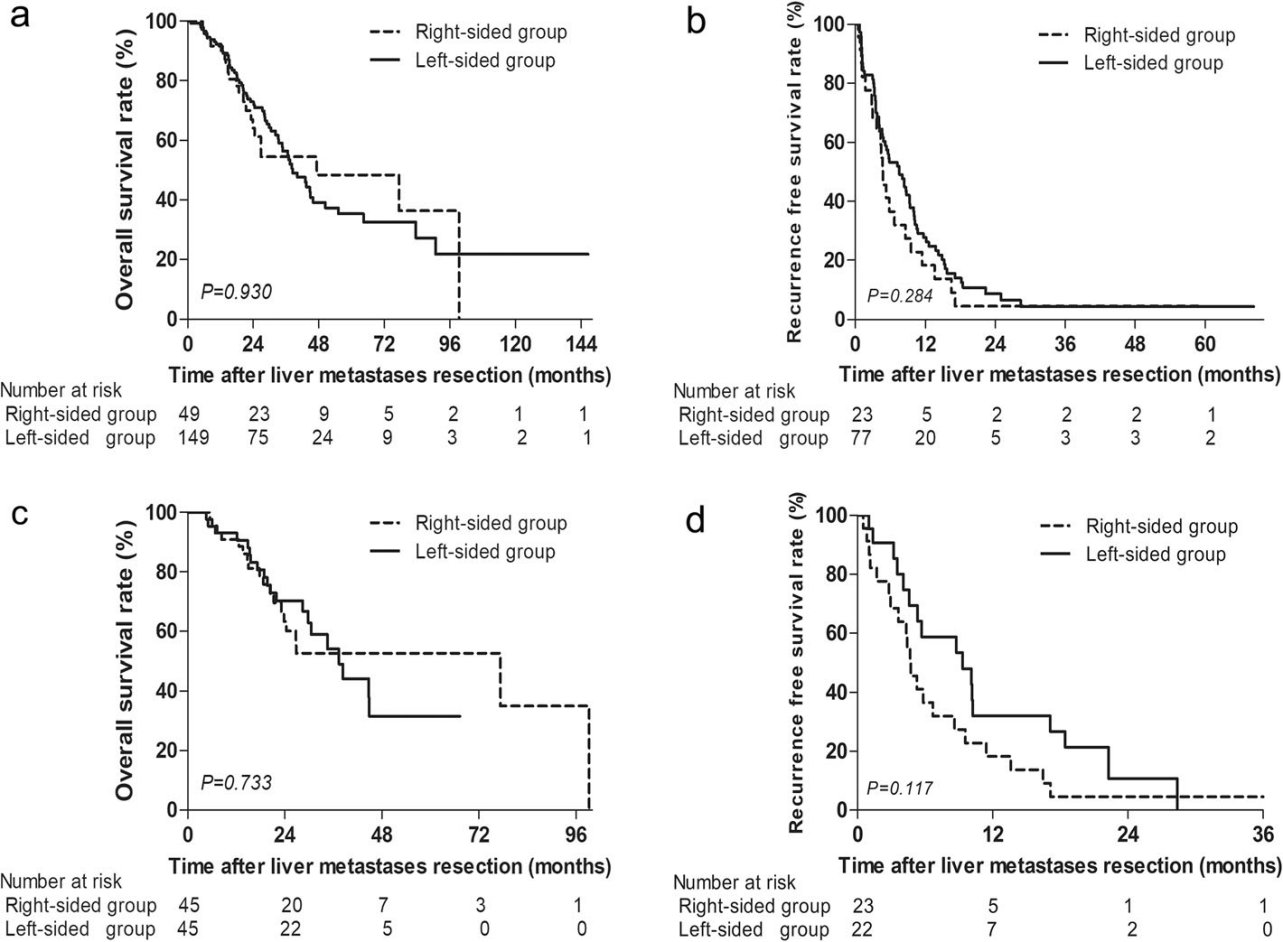
**a** Overall survival and **b** recurrence-free survival in CRLM patients stratified by CRC location with high CRS scores (score > 2). **c** Overall survival and **d** recurrence-free survival in CRLM patients stratified by CRC location with high CRS scores after PSM

We further explored the prognostic role of CRC location according to CRS scores and liver lesions. Similar OS was found in CRLM patients stratified by CRC location with different CRS scores. Significant worse RFS was found in the right-sided group before and after PSM among patients with low CRS scores (*P* = 0.037, *P* = 0.011, Fig. 2). However, RFS was comparable before and after PSM between the right-sided and left-sided group with high CRS scores (*P* = 0.284, *P* = 0.117; Fig. 3). Among patients with single liver lesion, OS and RFS were comparable before and after PSM between the right-sided and left-sided group (*P* = 0.322, *P* = 0.338; *P* = 0.191, *P* = 0.118; Supplementary Figure 2). Among patients with multiple liver metastases, significant worse RFS were also found in the right-sided group before and after PSM (*P* = 0.022, *P* = 0.012; Supplementary Figure 3b, 3d).

### Prognostic factors for patients after resection of liver metastases

Next, we performed univariate and multivariate analysis to identify prognostic factors in our patients. Factors including lymph node metastases (HR 1.600, 95% confidence interval [CI] 1.155–2.216, *P* = 0.005), liver lesions > 5 cm (HR 1.923, 95% CI 1.298-2.849, *P* = 0.001), and non-R0 resection (HR 1.998, 95% CI 1.424–2.804, *P* < 0.001) were found to affect OS (Table 2). For RFS, significant factors in multivariate analysis were tumor location (HR 0.659, 95% CI 0.478–0.910, *P* = 0.011), lymph node metastases (HR 1.533, 95% CI 1.159–2.029, *P* = 0.003), and resection combined with ablation (HR 1.793, 95% CI 1.253–2.566, *P* = 0.001) (Table 2**)**.

**Table 2:** Prognostic factors for overall survival and recurrence-free survival

Characteristics	Overall survival				Recurrence-free survival			
	Univariate	Multivariate analysis			Univariate	Multivariate analysis		
	***P*** value	HR	95%CI	***P*** value	***P*** value	HR	95%CI	***P*** value
Age (year), (≤ 55 vs. > 55)	0.477				0.786			
Gender (male vs. female)	0.785				0.479			
Tumor location								
Left-sided vs. right-sided	0.575				0.029*	0.659	0.478–0.910	0.011*
**Primary tumor characteristics**								
T stage (T3/T4 vs. T1/T2)	0.201				0.219			
N stage (N1/N2 vs. N0)	0.001*	1.600	1.155–2.216	0.005*	0.003*	1.533	1.159–2.029	0.003*
Tumor size (cm), (> 4 vs. ≤ 4)	0.254				0.628			
Postoperative chemotherapy (yes vs. no)	0.501				0.018*	1.417	0.944–2.217	0.093
**CRLM characteristics**								
Presentation of CRLM (synchronous vs. asynchronous)	0.766				0.190			
Preoperative chemotherapy (yes vs. no)	< 0.001*	1.279	0.898–1.822	0.172	0.001*	1.355	0.975–1.884	0.070
CEA (μg/L), (> 200 vs. ≤ 200)	0.020*	1.166	0.560–2.428	0.681	0.056			
CA19-9 (kU/L), (> 35 vs. ≤ 35)	0.015*	1.285	0.854–1.933	0.229	0.748			
Tumor size (cm), (> 5 vs. ≤ 5)	0.001*	1.923	1.298–2.849	0.001*	0.315			
Tumor number (multiple vs. single)	< 0.001*	1.446	0.988–2.117	0.058	<0.001*	1.244	0.907–1.706	0.176
Operative factors (with ablation vs. resection only)	< 0.001*	1.723	1.129–2.630	0.012	< 0.001*	1.793	1.253–2.566	0.001*
R0 resection (no vs. yes)	< 0.001*	1.998	1.424–2.804	< 0.001*				
Postoperative chemotherapy (yes vs. no)	0.057				0.319			

*HR* hazard ratio, *CI* confidence interval, *T stage* tumor stage, *N stage* node stage, *CRLM* colorectal liver metastases, *CEA* carcinoembryonic antigen, *CA 19-9* carbohydrate antigen 19-9, *R0 resection* hepatectomy on patients with clear histological margins

*P < 0.05

### Treatment of recurrence

Of the 611 patients, 240 (39.3%) patients had recurrence after liver metastases resection. There were 99 (39.0%) patients with relapsed after PSM. Treatment modality of recurrence in two groups is presented in Table 3. Before PSM, 41 (75.9%) and 119 (64.0%) patients underwent resection/ablation/chemotherapy alone, and 13 (24.1%) and 67 (36.0%) patients underwent combined therapy in the right-sided and left-sided group, respectively. Treatment modality of recurrence in two groups was comparable between two groups before and after PSM.

**Table 3:** Treatment of recurrence for patients after liver metastases resection

Characteristics	Before PSM (***n*** = 240)			After PSM (***n*** = 99)		
	Right-sided group (***n*** = 54)	Left-sided group (***n*** = 186)	***P*** value	Right-sided group (***n*** = 53)	Left-sided group (***n*** = 46)	***P*** value
Resection alone, *n* (%)	3 (5.6)	20 (10.7)	0.306	3 (5.7)	9 (19.6)	0.134
Ablation alone, *n* (%)	11 (20.4)	29 (15.6)	0.407	11 (20.8)	7 (15.2)	0.476
Chemotherapy alone, *n* (%)	27 (50.0)	70 (37.7)	0.103	18 (34.0)	17 (37.0)	0.756
Resection + chemotherapy, *n* (%)	3 (5.6)	9 (4.8)	0.735	3 (5.7)	2 (4.3)	1.000
Resection + radiotherapy, *n* (%)	1 (1.8)	3 (1.6)	0.905	1 (1.8)	0 (0.0)	NA
Ablation + chemotherapy, *n* (%)	3 (5.6)	25 (13.4)	0.149	3 (5.7)	2 (4.3)	1.000
Ablation + radiotherapy, *n* (%)	0 (0.0)	2 (1.1)	NA	0 (0.0)	0 (0.0)	NA
Radio + chemotherapy, *n* (%)	0 (0.0)	2 (1.1)	NA	0 (0.0)	0 (0.0)	NA
Supportive care, *n* (%)	6 (11.1)	26 (14.0)	0.585	14 (26.4)	9 (19.6)	0.421

*PSM* propensity score matching

## Discussion

Many clinicopathological factors and molecular features affect survival of CRC patients [16]. Among them, primary tumor location of CRC is a notable factor which can affect outcomes of patients [17]. So far, many evidence have revealed that the right-sided CRC patients have poorer prognosis than the left-sided CRC patients [18]. Differences in RAS status, microsatellite instability (MSI), and CpG island methylator (CIMP) phenotype may account for diverse clinicopathological characteristics and outcomes between the right-sided and left-sided CRC patients [19].

Recently, data from two pooled studies have shown that OS, progress free survival, and objective response rate were much worse among unresectable CRLM patients with right-sided tumor than those with left-sided tumor [20, 21]. However, whether primary tumor location of CRC affects prognosis of CRLM patients after hepatectomy remains debatable. One study found that CRLM patients with left-sided CRC have worse disease-free survival but better OS after liver resection, as authors suggested that tumors of patients with right-sided CRC relapsed less frequently than left-sided patients, but they had more aggressive disease once they recurred [22]. A meta-analysis concluded that CRLM patients with right-sided CRC had worse OS than those with left-sided CRC [15]. It should be noted that this analysis included nine non-Asian countries and only three studies from Asian countries. Some other studies showed that primary tumor location did not influence prognosis of CRLM patients after hepatectomy [23–25]. In CRLM patients after microwave ablation, comparable outcomes were also observed between the right-sided group and left-sided group [26, 27]. As such, whether primary tumor location of CRC affects prognosis of CRLM patients after hepatectomy remains debatable.

In our study, most baseline parameters such as largest size of liver tumors, number, and distribution of liver metastases were comparable between the two groups. However, selection bias might not have been completely avoided due to the retrospective nature of this study. By statistical adjustment, PSM is able to take full advantage of a large amount of data despite an observational design [28]. Application of PSM may have helped balance the underlying biases that were not analyzed in the study.

Compared with previous similar studies, our study has the following differences and new findings. First, the sample size of our study was larger, and PSM analysis was implemented to reduce the impact of selection bias on results. Second, as far as we know, unlike previous reports, our results show for the first time that the RFS in CRLM patients from the right-sided was poorer than those from the left-sided, but with no difference in OS. Third, we also conducted a further subgroup analysis to investigate the prognostic effects of the primary tumor location on patients with different CRS and tumor numbers. We found that with low CRS or multiple tumors, patients with CRLM from the right-sided had a higher recurrence rate than those from the left-sided. Overall, these novel findings suggest that patients with CRLM from the right-sided may need to receive intensive preoperative chemotherapy to eliminate micrometastatic disease and, more importantly, to further identify aggressive disease and select good candidates for surgery. Additionally, the benefit of surgery and high risk of recurrence should be carefully taken into consideration. On the other hand, less invasive non-surgical strategies for small liver lesions such as radiofrequency ablation or stereotactic body radiotherapy (SBRT) might be an effective alternative to resection as the first-line treatment. It should be noted that subsequent treatments are crucial for prolonging survival of patients after recurrence. Thus, more frequent follow-up after surgery to detect early recurrence may help improve the prognosis of these patients.

Compared to the CRLM from the left-sided, the more aggressive tumor behavior of the CRLM from the right-sided may contribute to its worse RFS. Early studies have shown that tumors in the right colon were larger, more often poorly differentiated and more often had a peritumoral lymphocytic infiltrate than tumors in the left colon and rectum [10]. Besides, right-sided CRC was more characterized by high MSI (MSI-H) and more BRAF mutations [29]. Many studies demonstrated that right-sided CRC patients presented with a significantly worse survival than those with left-sided CRC [11, 30]. As there is a high concordance of molecular characteristics between primary CRC and their corresponding liver metastases, even after R0 resection, aggressive biological behavior may lead to shorter time to recurrence in CRLM patients from the right-sided [31]. The disparity between the results of RFS and OS may be due to benefit of subsequent therapies after recurrence. Tumors may recur more frequently in CRLM patients with right-sided CRC, but efficient and multi-discipline therapies to treat recurrence lesions may result in comparable prognosis.

The inconsistent effects of primary tumor location in CRC and CRLM patients may be partially explained by the following reasons. Firstly, there were studies reported that prognosis was much worse among unresectable CRLM patients from right-sided CRC than those from left-sided CRC [20, 21]. Therefore, there might be more patients with unresectable CRLM and/or extrahepatic disease which were unable to convert to resectable disease from right-sided CRC than from left-sided CRC. These unresectable patients accounted for the majority of population and had a greater impact on the overall prognosis. Thus, as indicated in our study and other studies, although the results showed that the OS were comparable in patients with resectable CRLM from right-sided and left-sided CRC, these are not contradictory to previous reports that right-sided CRC patients had worse OS than left-sided CRC patients. Secondly, since resection of liver metastases is the potentially curative approach for CRLM patients, the benefits of hepatectomy may neutralize the prognostic effect of primary tumor location for CRLM patients [24, 25]. Thirdly, the prognostic value of primary tumor location may depend on tumor stages. There were evidences that survival was not affected by tumor location in early stage CRC patient while it was influenced in patients with advanced unresectable CRLM [18, 32, 33]. Moreover, difference in ethnicity may also contribute to the discrepancy in result. Therefore, the prognostic value of tumor location needs further prospective investigation.

It is important to note the limitations in our study. Although PSM was used to reduce selection bias caused by retrospective design, our study only included patients in a single institution. Furthermore, genetic information such as RAS type and BRAF type were not available in most of our patients. We were unable to assess the prognostic impact of genetic status in two groups. Hence, future studies which include multicenter, large scale of patients with genetic data are needed to confirm our conclusion.

## Conclusion

Compared to patients with left-sided primary tumors, patients with right-sided primary tumors had a worse RFS but similar OS. Careful preoperative evaluation, intensive preoperative chemotherapy and frequent follow-up to detect early recurrence might be justified for CRLM patients with right-sided primary tumors.

## Supplementary information

**Additional file 1: Supplementary Figure 1.** Histograms with overlaid kernel density estimates for standardized mean differences before and after PSM. The graph shows the reduction of imbalance after the matching process between right-sided and left-sided group patients. **Supplementary Figure 2.** (a) Overall survival and (b) recurrence free survival in CRLM patients stratified by CRC location with single liver lesion. (c) Overall survival and (d) recurrence free survival in CRLM patients stratified by CRC location with single liver lesion after PSM. **Supplementary Figure 3.** (a) Overall survival and (b) recurrence free survival in CRLM patients stratified by CRC location with multiple liver lesions. (c) Overall survival and (d) recurrence free survival in CRLM patients stratified by CRC location with multiple liver lesions after PSM.

## Data Availability

The datasets generated during and/or analyzed during the current study are available from the corresponding author on reasonable request.

## Abbreviations

CRC: Colorectal cancer
CRLM: Colorectal liver metastases
PSM: Propensity score analysis
OS: Overall survival
RFS: Recurrence-free survival
CRS: Clinical risk score
CEA: Carcinoembryonic antigen
ECOG-PS: Eastern Cooperative Oncology Group-performance status
MRI: Magnetic resonance imaging
CT: Computed tomography
US: Ultrasonography
SMD: Standardized mean differences
HR: Hazard ratio
CI: Confidence interval
MSI: Microsatellite instability
CIMP: CpG island methylator
SBRT: Stereotactic body radiotherapy
