# Supplementary material for: Primary tumor location affects recurrence-free survival for patients with colorectal liver metastases after hepatectomy: a propensity score matching analysis
Source: World J Surg Oncol. 2020 May 18;18:98. doi: 10.1186/s12957-020-01875-y (PMC7236531; doi:10.1186/s12957-020-01875-y)
Supplement: Supplementary file 1 — Additional file 1: Supplementary Figure 1. Histograms with overlaid kernel density estimates for standardized mean differences before and after PSM. The graph shows the reduction of imbalance after the matching process between right-sided and left-sided group patients. Supplementary Figure 2. (a) Overall survival and (b) recurrence free survival in CRLM patients stratified by CRC location with single liver lesion. (c) Overall survival and (d) recurrence free survival in CRLM patients stratified by CRC location with single liver lesion after PSM. Supplementary Figure 3. (a) Overall survival and (b) recurrence free survival in CRLM patients stratified by CRC location with multiple liver lesions. (c) Overall survival and (d) recurrence free survival in CRLM patients stratified by CRC location with multiple liver lesions after PSM. [file 12957_2020_1875_MOESM1_ESM.pdf]

Supplementary material

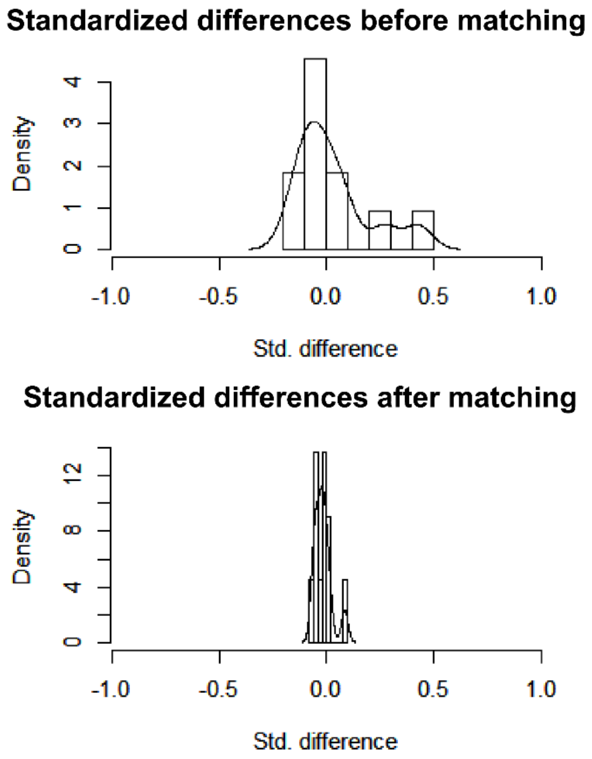

Supplementary Figure 1. Histograms with overlaid kernel density estimates for standardized mean differences before and after PSM. The graph shows the reduction of imbalance after the matching process between right-sided and left-sided group patients.

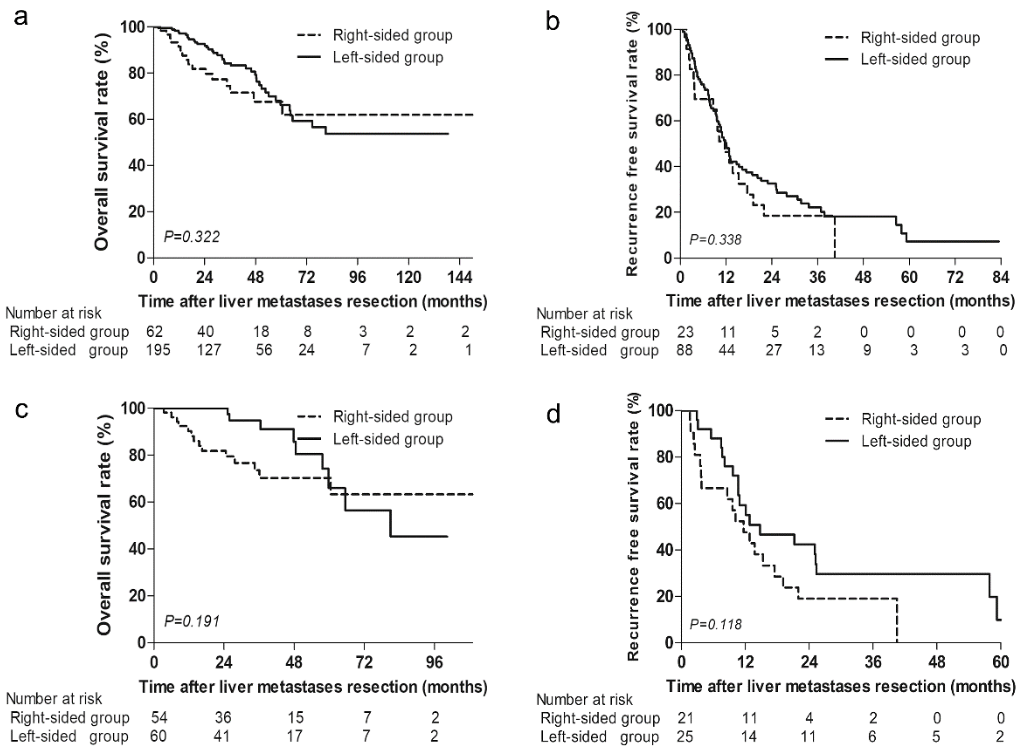

Supplement Figure 2. (a) Overall survival and (b) recurrence free survival in CRLM patients stratified by CRC location with single liver lesion. (c) Overall survival and (d) recurrence free survival in CRLM patients stratified by CRC location with single liver lesion after PSM.

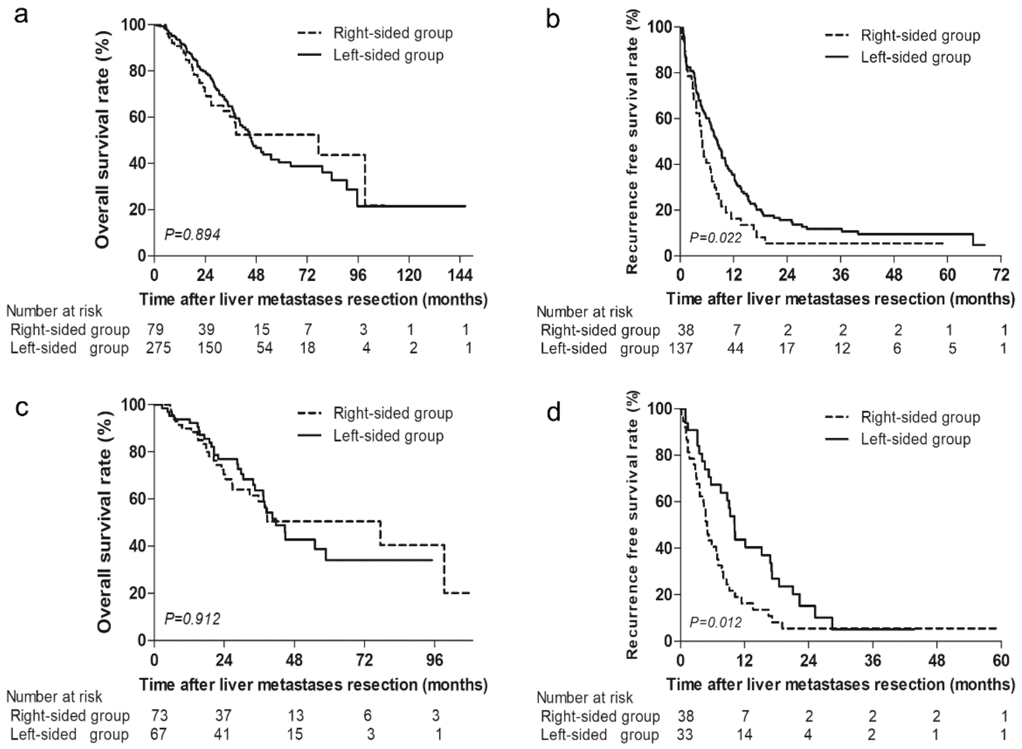

Supplement Figure 3. (a) Overall survival and (b) recurrence free survival in CRLM patients stratified by CRC location with multiple liver lesions. (c) Overall survival and (d) recurrence free survival in CRLM patients stratified by CRC location with multiple liver lesions after PSM.
